# Multicenter Study of Controlling Nutritional Status (CONUT) Score as a Prognostic Factor in Patients With HIV-Related Renal Cell Carcinoma

**DOI:** 10.3389/fimmu.2021.778746

**Published:** 2021-11-30

**Authors:** Wenrui Xue, Yu Zhang, Hua Wang, Yu Zhang, Xiaopeng Hu

**Affiliations:** ^1^Beijing Youan Hospital, Capital Medical University, Beijing, China; ^2^Beijing Ditan Hospital, Capital Medical University, Beijing, China; ^3^Chengdu Public Health Clinical Medical Center, Sichuan, China; ^4^Beijing Chaoyang Hospital, Capital Medical University, Beijing, China

**Keywords:** controlling nutritional status score, HIV-related renal cell carcinoma, prognostic factor, cancer-specific survival (CSS), overall survival (OS), disease-free survival (DFS), highly active antiretroviral therapy (HAART)

## Abstract

**Objective:**

In recent years, the controlled nutritional status (CONUT) score has been widely recognized as a new indicator for assessing survival in patients with urological neoplasms, including renal, ureteral, and bladder cancer. However, the CONUT score has not been analyzed in patients with HIV-related urological neoplasms. Therefore, we aimed to evaluate the prognostic significance of the CONUT score in patients with HIV-related renal cell carcinoma (RCC).

**Methods:**

A total of 106 patients with HIV-related RCC were recruited from four hospitals between 2012 and 2021, and all included patients received radical nephrectomy or partial nephrectomy. The CONUT score was calculated by serum albumin, total lymphocyte counts, and total cholesterol concentrations. Patients with RCC were divided into two groups according to the optimal cutoff value of the CONUT score. Survival analysis of different CONUT groups was performed by the Kaplan–Meier method and a log rank test. A Cox proportional risk model was used to test for correlations between clinical variables and cancer-specific survival (CSS), overall survival (OS), and disease-free survival (DFS). Clinical variables included age, sex, hypertension, diabetes, tumor grade, Fuhrman grade, histology, surgery, and CD4+ T lymphocyte count.

**Result:**

The median age was 51 years, with 93 males and 13 females. At a median follow-up of 41 months, 25 patients (23.6%) had died or had tumor recurrence and metastasis. The optimal cutoff value for the CONUT score was 3, and a lower CONUT score was associated with the Fuhrman grade (P=0.024). Patients with lower CONUT scores had better CSS (HR 0.197, 95% CI 0.077-0.502, P=0.001), OS (HR 0.177, 95% CI 0.070-0.446, P<0.001) and DFS (HR 0.176, 95% CI 0.070-0.444, P<0.001). Multivariate Cox regression analysis indicated that a low CONUT score was an independent predictor of CSS, OS and DFS (CSS: HR=0.225, 95% CI 0.067-0.749, P=0.015; OS: HR=0.201, 95% CI 0.061-0.661, P=0.008; DFS: HR=0.227, 95% CI 0.078-0.664, P=0.007). In addition, a low Fuhrman grade was an independent predictor of CSS (HR 0.192, 95% CI 0.045-0.810, P=0.025), OS (HR 0.203, 95% CI 0.049-0.842, P=0.028), and DFS (HR 0.180, 95% CI 0.048-0.669, P=0.010), while other factors, such as age, sex, hypertension, diabetes, tumor grade, histology, surgery, and CD4+ T lymphocyte count, were not associated with survival outcome.

**Conclusion:**

The CONUT score, an easily measurable immune-nutritional biomarker, may provide useful prognostic information in HIV-related RCC.

## Introduction

RCC is the most common pathologic type of renal cancer and the seventh most common tumor, accounting for 2% to 3% of all cancers (1). Twenty percent of newly diagnosed RCC patients have advanced disease, and approximately 30% experience local or distant disease recurrence after surgery for localized RCC (2). In recent years, relatively few infection cases of HIV-related RCC have been reported worldwide. Patients with such RCC have concurrent immune infection. Human immunodeficiency virus (HIV) infects human dendritic cells and macrophages and activates CD4+ T lymphocytes, resulting in disruption of the immune system, so the tumor incidence and mortality differ from those in ordinary RCC patients (3). RCC is more common in HIV-infected individuals than in age-matched non-HIV-infected individuals and is a common cause of morbidity and mortality. Possible mechanisms for this increased risk include reduced immune surveillance, direct effects of viral proteins, or cytokine dysregulation (4, 5). With the widespread application of and tremendous progress in early activation of highly active antiretroviral therapy (HAART), both virological suppression and immune recovery in patients with HIV-related RCC have been maintained at a good level (6). However, non-AIDS-defining cancers (non-ADCs), including urinary cancers, anal cancers, lung cancers, breast cancers and skin cancers, are still three times more frequent (7). If patients with HIV-related RCC can be assessed early, their survival could be significantly improved. Therefore, it is important to develop off-the-shelf biomarkers that can predict and even modify tumor outcomes based on risk stratification (8).

Immunological status comprising inflammatory and nutritional status, remains an important predictor of prognosis in patients with malignant tumors (9). Several biomarkers, such as the prognostic nutritional index (PNI) and the neutrophil to lymphocyte ratio (NLR), have been reported to be independent prognostic factors (10–12). Recently, the CONUT score, which is calculated from serum albumin, total lymphocyte counts, and total cholesterol concentration, has gained attention as a biomarker for predicting survival in patients with multiple cancers. A high CONUT score means lower levels of albumin, lymphocytes, and cholesterol, which are often associated with poorer nutritional and immune status in patients and may lead to poorer survival (13). Maintaining optimal nutritional status can greatly improve quality of life while reducing comorbidities, progression of HIV infection, and HIV-related mortality (14, 15). In addition, good nutrition also helps HIV-infected patients absorb HIV drugs (16). The effects of poor nutritional status and HIV are synergistic and interrelated, thus amplifying their respective harmful effects (15, 17). Increasing evidence suggests that, in addition to the genetic basis, host nutritional status and inflammatory responses also play an important role in cancer development and progression (18). At present, some articles suggest that the CSS, OS and DFS of patients with 5-year ordinary RCC (non-HIV related) in the low-CONUT group are significantly higher than those in the high-CONUT group (8, 19–24), but some articles suggest that a high CONUT score is not related to the prognosis of patients with ordinary RCC (25). However, there are no articles about the relationship between CONUT score and HIV-related RCC. To the best of our knowledge, this is the first multicenter study to evaluate the prognostic value of the CONUT score in HIV-related RCC.

## Materials and Methods

### Patients

We performed an open-label, retrospective, multicenter, cohort study. A total of 106 patients with HIV-related RCC who underwent radical nephrectomy or partial nephrectomy were included. All participants underwent preoperative urological CT examination showing a renal carcinoma volume ≤7 cm between 2012 and 2021. We excluded patients with ordinary RCC without HIV infection, patients with lymph node metastasis or distant metastasis, and patients with no follow-up results. All enrolled patients had provided blood samples with results for serum albumin, total lymphocyte counts, and total cholesterol concentration one week before surgery and were treated with HAART and monitored for associated CD4+ T lymphocyte count.

Pathological stage was determined according to the 2010 TNM grade and tumor grade according to the Fuhrman grading system. This study was in accordance with the Helsinki Declaration and approved by the Ethics Review Committee of all included hospitals. During follow-up, patients or their next of kin were informed of the study in detail, and verbal consent was obtained. All data are kept confidential.

### Follow-Up

Every three months within the first 3 years after surgery, the patient was admitted to the outpatient department of the hospital for routine blood examination, blood biochemistry, chest X-ray, abdominal color Doppler ultrasound and enhanced urinary CT examination. After 3 years, the above review was performed every 6 months until tumor recurrence, metastasis or death. Relapse is equal to the first detection of local recurrence, and metastasis is equal to the first discovery of lymph node or distant organ metastases (lung metastasis, brain metastasis, liver metastasis, etc.). Death was confirmed by relevant information from the hospitals or notification by the patient’s family during telephone follow-up.

### Study Endpoints

We considered CSS, OS, and DFS as the end points of the study (in months). CSS was defined as the time from the date of surgery to cancer-related death. OS was defined as the time from the date of surgery to the death of the individual from any cause. DFS was defined as the time from the date of surgery to radiologically or histologically confirmed recurrence or metastasis.

### CONUT Score, PNI and NLR

The CONUT score was calculated by serum albumin, total lymphocyte counts, and total cholesterol concentration (**Table 1**). The optimal cutoff value of the CONUT score was determined using the receiver operating characteristic (ROC) curve and the maximum Youden index value. The PNI was calculated as 10 × serum albumin (g/dl) + 0.005 × total lymphocyte count (per mm3). The NLR was calculated as the ratio of the number of neutrophils to the number of lymphocytes.

**Table 1:** Definition of CONUT score.

Parameters	CONUT			
	Normal	Light	Moderate	Severe
Serum albumin (g/dL)	≥3.50	3.00-3.49	2.50-2.99	<2.50
Score	0	2	4	6
Total lymphocyte (/mm^3^)	≥1600	1200-1599	800-1199	<800
Score	0	1	2	3
Total cholesterol (mg/dL)	≥180	140-179	100-139	<100
Score	0	1	2	3
CONUT score (total)	0-1	2-4	5-8	9-12

### Statistics

A chi-square test was used to analyze the correlations between the CONUT score and variables including age, sex, hypertension, diabetes, tumor grade, Fuhrman grade, histology, surgery, and CD4+ T lymphocyte count. Kaplan–Meier survival curves were plotted to estimate CSS, OS, and DFS. The predictors of CSS, OS and DFS were determined by univariate analysis, a Cox proportional risk model was used for multivariate analysis evaluation, and variables with P<0.05 in univariate analysis were included in subsequent multivariate analysis. GraphPad Prism Version 9 (GraphPad Software, La Jolla California USA, www.graphpad.com) was used to generate survival curves. Statistical analysis and ROC curves mapping were performed using SPSS version 23 (SPSS Inc., Chicago, IL, USA).

## Results

### CONUT Score and Its Cutoff Value

**Figure 1:**
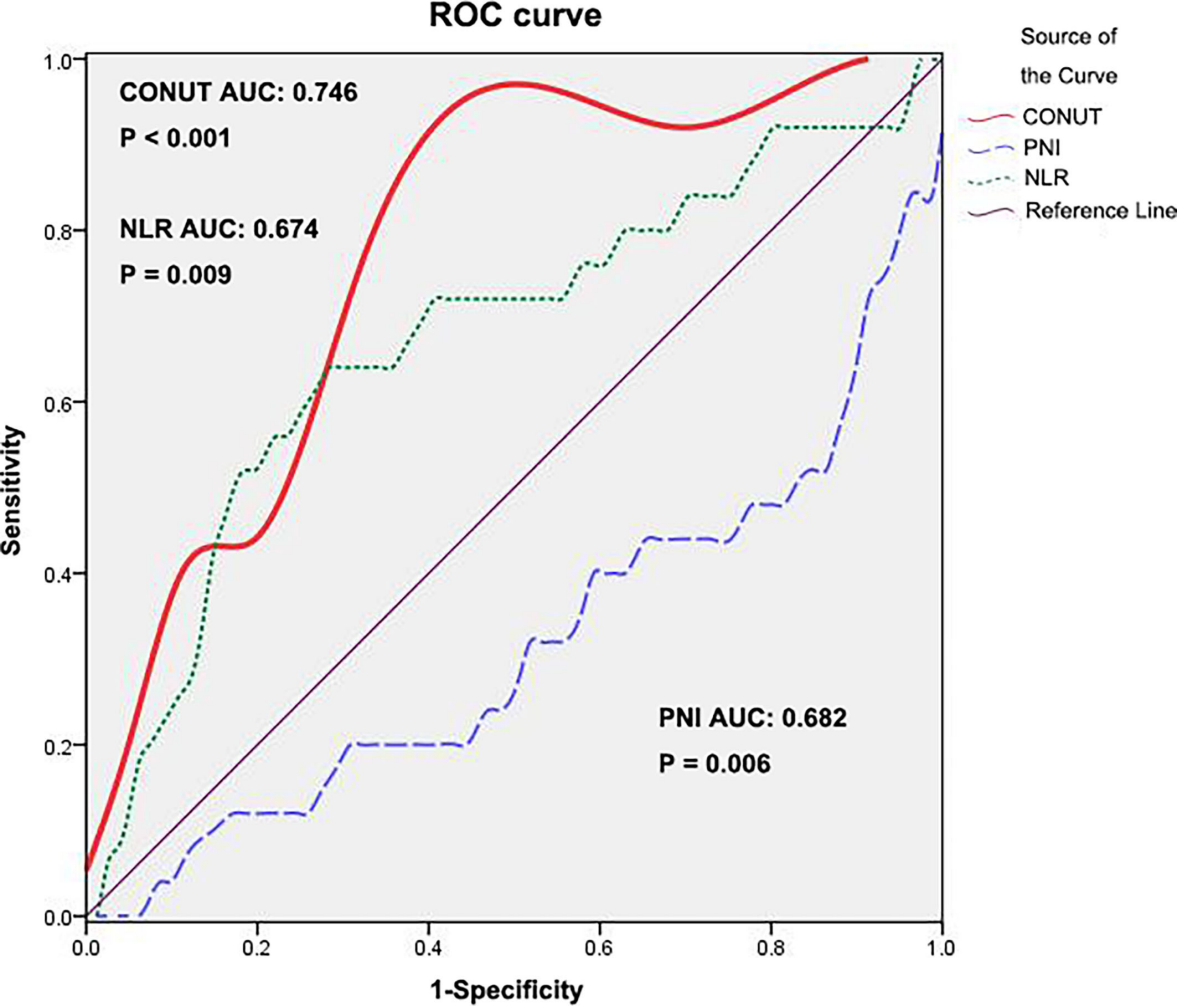
ROC curve for CONUT, PNI and NLR. ROC, receiver operating characteristic; CONUT, controlling nutritional status; PNI, prognostic nutritional index; NLR, neutrophil–lymphocyte ratio.

According to ROC analysis, the Youden index was used to determine the optimal cutoff value of the CONUT score as 3 (AUC: 0.746, 95% CI: 0.638-0.855, P<0.001, **Figure 1**. The area under the receiver operating characteristics curve, AUC). The CONUT score was assessed by dichotomous variables (low: <3, high: ≥3).

### Clinicopathological Features

Of the 106 patients enrolled, with a median age of 51 (IQR 27-75) years at the time of surgery, 93 were males, 13 were females, 80 underwent radical nephrectomy, and 26 underwent partial nephrectomy. Among them, 104 cases were clear cell carcinoma, 87 cases were T1N0M0, and 19 cases were T3N0M0. For the Fuhrman classification, 65 were grades I-II, and 41 were grades III-IV. The median CD4+ T lymphocyte count value was 435 (IQR 48-1536) cells/µl. The highest preoperative viral load was 1,018,049 copies/mL, and the lowest was undetectable (**Table 2**). The CONUT score was high in 45 cases (42%) and low in 61 cases (58%), and was closely correlated with the Fuhrman grade. A low CONUT score was significantly associated with lower Fuhrman grade (I-II vs III-IV, 66.2% vs 43.9%, respectively, p=0.024). The CONUT score had no significant correlation with age, sex, hypertension, diabetes, tumor grade, histology, surgery, or CD4+ T lymphocyte count (P**>**0.05) (**Table 3**).

**Table 2:** Basic information of the cases and HIV- related data.

Case	Age	Gender	Fuhr-man	Tumor grade	Histology	Comorbidity	CD4 count (cells/ul)	Viral load (copies/ml)	Surgery	CONUT	PNI	NLR
1	59	Male	III-IV	T1N0M0	Clear cell	Hypertension	614	NT	RN	1	54.75	0.9628
2	74	Male	I-II	T1N0M0	Clear cell	Hypertension	356	NT	RN	2	41.65	2.7287
3	43	Male	I-II	T1N0M0	Clear cell	None	400	TND	RN	2	47.65	1.6163
4	58	Male	I-II	T1N0M0	Clear cell	None	1536	341	RN	5	45.75	2.3498
5	54	Male	III-IV	T1N0M0	Clear cell	Hypertension	628	NT	RN	1	52.75	1.7355
6	51	Male	I-II	T1N0M0	Clear cell	Diabetes	291	TND	RN	1	52.3	2.5505
7	54	Male	I-II	T1N0M0	Clear cell	Hypertension+ Diabetes	880	TND	PN	0	58.05	1.0433
8	51	Male	III-IV	T3N0M0	Clear cell	None	190	6495	RN	2	49.4	1.9253
9	34	Male	III-IV	T3N0M0	Clear cell	None	462	TND	RN	3	49.55	2.9802
10	72	Male	III-IV	T1N0M0	Clear cell	None	378	NT	RN	2	47.65	3.6067
11	54	Female	III-IV	T1N0M0	Clear cell	None	535	63188	RN	1	48.65	1.4571
12	51	Male	I-II	T1N0M0	Clear cell	None	142	409601	RN	1	47.25	0.8667
13	53	Male	I-II	T1N0M0	Non-clear cell	Hypertension	328	31164	RN	0	52.85	2.0675
14	55	Male	III-IV	T1N0M0	Clear cell	None	420	<40	RN	1	49.1	2.8654
15	39	Male	I-II	T1N0M0	Clear cell	Hypertension	190	TND	RN	0	47.65	1.5698
16	46	Male	I-II	T1N0M0	Clear cell	Hypertension	267	61	RN	0	52.5	3.0571
17	67	Male	III-IV	T1N0M0	Clear cell	Diabetes	821	TND	RN	2	43.6	4.2982
18	59	Male	III-IV	T1N0M0	Clear cell	None	375	TND	RN	3	52.25	2.8956
19	56	Male	I-II	T3N0M0	Clear cell	None	495	TND	PN	2	51.5	3.0782
20	54	Female	I-II	T1N0M0	Clear cell	None	687	552	RN	0	55.25	1.9875
21	62	Male	III-IV	T1N0M0	Clear cell	Hypertension	325	NT	RN	3	40.5	3.8593
22	49	Male	III-IV	T1N0M0	Clear cell	None	641	<40	RN	1	51.25	0.9889
23	66	Male	I-II	T1N0M0	Clear cell	Diabetes	531	180	RN	2	44.25	2.9136
24	30	Female	I-II	T1N0M0	Clear cell	None	604	TND	PN	2	51.25	1.0404
25	39	Male	I-II	T1N0M0	Clear cell	None	296	NT	RN	1	53.8	0.9825
26	51	Male	III-IV	T1N0M0	Clear cell	None	151	73907	RN	3	45.85	1.2980
27	50	Male	I-II	T1N0M0	Clear cell	None	327	4909	RN	2	48.95	2.7248
28	50	Male	III-IV	T1N0M0	Clear cell	None	665	TND	RN	2	55.1	1.1713
29	48	Male	III-IV	T1N0M0	Clear cell	None	589	TND	PN	2	52.15	1.2731
30	57	Male	III-IV	T1N0M0	Clear cell	Diabetes	1062	<40	RN	0	60.9	0.8381
31	52	Male	I-II	T1N0M0	Clear cell	Hypertension	268	TND	RN	0	56.9	1.1234
32	36	Male	I-II	T1N0M0	Clear cell	Diabetes	140	174	RN	3	46.75	3.7321
33	32	Male	I-II	T1N0M0	Clear cell	None	1229	TND	RN	3	47.95	6.4360
34	51	Male	III-IV	T3N0M0	Clear cell	None	589	TND	RN	2	54.9	3.0312
35	65	Male	III-IV	T1N0M0	Clear cell	None	237	2241	RN	5	41.3	3.7697
36	50	Male	III-IV	T3N0M0	Clear cell	None	179	57922	RN	2	41.4	3.3089
37	27	Male	I-II	T1N0M0	Clear cell	None	1082	TND	PN	2	44.3	4.0905
38	43	Female	I-II	T1N0M0	Clear cell	None	538	TND	PN	6	37	5.0417
39	46	Male	I-II	T3N0M0	Clear cell	None	567	<40	RN	2	45.65	1.1357
40	63	Male	III-IV	T3N0M0	Clear cell	None	73	1018049	RN	8	32.6	5.0417
41	51	Male	I-II	T1N0M0	Clear cell	None	537	77565	RN	1	54.6	1.2692
42	35	Male	I-II	T1N0M0	Clear cell	None	223	TND	PN	2	46.15	1.3043
43	52	Male	I-II	T1N0M0	Clear cell	None	321	14317	PN	3	52.3	1.6026
44	61	Male	III-IV	T3N0M0	Clear cell	None	148	34900	RN	7	36.28	2.5641
45	41	Male	I-II	T1N0M0	Clear cell	None	48	59900	RN	4	44.55	1.5039
46	55	Male	I-II	T1N0M0	Clear cell	Diabetes	438	TND	PN	4	43.25	2.4747
47	47	Male	I-II	T1N0M0	Clear cell	None	530	TND	PN	2	45.1	1.2083
48	54	Female	I-II	T1N0M0	Clear cell	None	234	TND	RN	6	38.1	2.6764
49	61	Female	I-II	T1N0M0	Clear cell	None	311	TND	RN	4	40.85	3.0689
50	75	Male	I-II	T1N0M0	Clear cell	Hypertension	180	TND	PN	5	45.55	3.0093
51	67	Male	I-II	T1N0M0	Non-clear cell	None	356	TND	RN	4	40.25	3.3926
52	57	Male	III-IV	T1N0M0	Clear cell	Hypertension+ Diabetes	1399	TND	RN	1	49.05	1.4150
53	45	Male	III-IV	T3N0M0	Clear cell	None	530	TND	RN	6	38.7	5.0176
54	39	Male	I-II	T1N0M0	Clear cell	None	335	TND	PN	3	39.3	4.6872
55	63	Male	I-II	T1N0M0	Clear cell	None	423	4528	RN	2	46.5	2.9630
56	41	Male	III-IV	T1N0M0	Clear cell	None	741	TND	RN	5	37.27	4.9724
57	48	Male	I-II	T1N0M0	Clear cell	None	552	<40	RN	1	47.5	2.5657
58	68	Male	III-IV	T1N0M0	Clear cell	None	269	TND	PN	2	50.9	3.0171
59	46	Female	III-IV	T3N0M0	Clear cell	None	412	TND	RN	2	50.25	1.5623
60	36	Male	III-IV	T1N0M0	Clear cell	None	637	380	RN	6	32.28	3.9743
61	55	Male	I-II	T3N0M0	Clear cell	None	190	TND	RN	2	43.9	3.0587
62	38	Male	III-IV	T1N0M0	Clear cell	None	423	TND	RN	3	46.9	3.2568
63	64	Male	III-IV	T1N0M0	Clear cell	None	449	900	RN	5	41.7	3.4102
64	69	Male	I-II	T1N0M0	Clear cell	None	432	TND	RN	1	52.15	3.8576
65	51	Male	III-IV	T3N0M0	Clear cell	None	547	82080	RN	2	45.2	1.4568
66	49	Female	I-II	T1N0M0	Clear cell	None	734	TND	RN	3	41.15	3.7596
67	38	Male	I-II	T1N0M0	Clear cell	None	353	TND	PN	2	43.9	4.3264
68	55	Male	I-II	T1N0M0	Clear cell	None	229	24611	PN	2	50.4	2.6384
69	46	Male	I-II	T1N0M0	Clear cell	None	431	TND	PN	3	39.9	4.7919
70	61	Female	I-II	T1N0M0	Clear cell	None	524	TND	RN	5	39.4	4.5244
71	61	Male	III-IV	T1N0M0	Clear cell	None	421	<40	RN	5	38.43	3.1028
72	49	Male	I-II	T3N0M0	Clear cell	None	778	<40	PN	3	49.8	2.0970
73	33	Male	I-II	T1N0M0	Clear cell	Diabetes	567	TND	PN	4	36.8	4.5654
74	64	Male	I-II	T3N0M0	Clear cell	None	258	3600	RN	6	38.75	4.1971
75	57	Male	I-II	T1N0M0	Clear cell	None	655	TND	RN	4	46.05	3.2347
76	54	Male	I-II	T1N0M0	Clear cell	None	356	TND	RN	1	48.1	1.6593
77	42	Male	I-II	T1N0M0	Clear cell	None	551	<40	RN	2	45.6	2.6567
78	45	Male	I-II	T1N0M0	Clear cell	None	332	TND	RN	1	46.65	3.0105
79	53	Male	III-IV	T3N0M0	Clear cell	None	533	TND	RN	6	40.6	3.5971
80	66	Male	I-II	T1N0M0	Clear cell	None	550	TND	PN	3	42.9	3.1775
81	41	Female	I-II	T1N0M0	Clear cell	Diabetes	477	TND	RN	2	45.4	2.8861
82	45	Male	I-II	T1N0M0	Clear cell	None	377	TND	RN	3	48.05	1.2693
83	55	Male	III-IV	T1N0M0	Clear cell	None	636	5352	RN	2	43.75	2.2327
84	70	Male	III-IV	T3N0M0	Clear cell	Hypertension	359	TND	RN	3	48.1	2.4647
85	48	Male	III-IV	T3N0M0	Clear cell	None	678	TND	RN	2	45.15	3.6537
86	50	Male	I-II	T1N0M0	Clear cell	None	790	TND	RN	1	48.2	2.6974
87	44	Male	I-II	T1N0M0	Clear cell	None	358	TND	RN	2	48	1.2387
88	29	Male	I-II	T1N0M0	Clear cell	None	559	NT	PN	2	48.3	3.4697
89	68	Male	I-II	T1N0M0	Clear cell	None	243	TND	RN	5	39.15	4.6874
90	49	Female	I-II	T1N0M0	Clear cell	None	438	TND	PN	1	44.55	2.5813
91	38	Male	III-IV	T1N0M0	Clear cell	None	457	<40	RN	7	36.6	4.6687
92	51	Male	I-II	T1N0M0	Clear cell	None	221	TND	RN	4	39.45	4.4367
93	33	Male	I-II	T1N0M0	Clear cell	Hypertension	573	56781	RN	3	45.95	2.1187
94	67	Male	III-IV	T1N0M0	Clear cell	None	329	TND	RN	3	39.45	4.7652
95	56	Male	I-II	T1N0M0	Clear cell	None	343	2538	RN	2	47.25	1.1495
96	48	Male	I-II	T1N0M0	Clear cell	None	431	TND	PN	1	49.45	2.7465
97	31	Male	III-IV	T1N0M0	Clear cell	None	415	TND	RN	5	39.75	3.9853
98	44	Male	I-II	T1N0M0	Clear cell	None	227	<40	PN	1	50.3	1.0147
99	61	Male	I-II	T3N0M0	Clear cell	None	621	44090	RN	2	45.95	2.6584
100	56	Female	I-II	T1N0M0	Clear cell	None	591	TND	PN	4	38.9	4.3251
101	52	Male	III-IV	T1N0M0	Clear cell	None	790	NT	RN	2	45.45	2.7892
102	44	Male	I-II	T1N0M0	Clear cell	None	544	TND	PN	3	40.3	3.4663
103	51	Male	III-IV	T1N0M0	Clear cell	Diabetes	980	<40	RN	2	44.3	2.6835
104	38	Male	III-IV	T1N0M0	Clear cell	None	555	399	RN	5	41.15	3.4678
105	57	Male	III-IV	T3N0M0	Clear cell	None	288	TND	RN	2	49.15	0.9785
106	37	Female	I-II	T1N0M0	Clear cell	Hypertension	543	TND	PN	1	48	2.4318

NT, not tested; TND, target not detected; RN, radical nephrectomy; PN, partial nephrectomy; CONUT, controlling nutritional status; PNI, prognostic nutritional index; NLR, neutrophil–lymphocyte ratio.

**Table 3:** Clinicopathological characteristics of the 106 patients according to different CONUT groups.

Factors	Total (n = 106)	CONUT<3 (n = 61)	CONUT≥3(n = 45)	*P*-value*
**Age (years)**				
≤65	94	55	39	*P*=0.574
>65	12	6	6	
**Gender**				
Male	93	54	39	*P*=0.773
Female	13	7	6	
**Hypertention**				
Yes	14	10	4	*P*=0.259
No	92	51	41	
**Diabetes**				
Yes	11	8	3	*P*=0.451
No	95	53	42	
**Tumor grade**				
T1N0M0	87	50	37	*P*=0.973
T3N0M0	19	11	8	
**Fuhrman grade**				
I-II	65	43	22	***P*=0.024**
III-IV	41	18	23	
**Histology**				
Clear cell	104	60	44	*P*=0.671
Non-clear cell	2	1	1	
**Surgery**				
RN	80	46	34	*P*=0.986
PN	26	15	11	
**CD4+ T lymphocyte count (cells/ul)**				
≥200	95	56	39	*P*=0.593
<200	11	5	6	

*Chi-square test. Bold value indicates statistical significance in univariate and multivariate analysis which had been detailed in the “Results” section.

RN, radical nephrectomy; PN, partial nephrectomy.

### Survival Outcome

**Figure 2:**
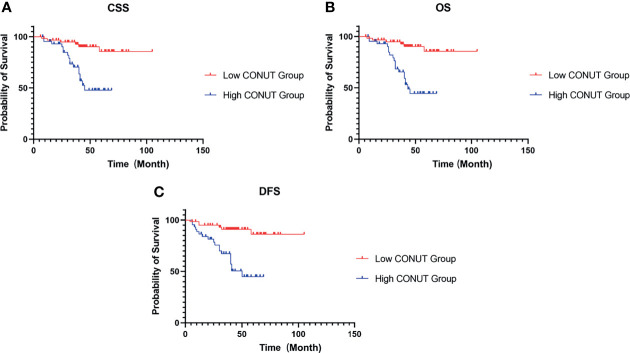
Kaplan–Meier survival curves for HIV-related RCC patients treated with surgery. Survival curves set at cutoff value 3 for CSS **(A)**, OS **(B)** and DFS **(C)**. RCC, renal cell carcinoma; CSS, cancer-specific survival; OS, overall survival; DFS, disease-free survival; CONUT, controlling nutritional status.

The median postoperative follow-up time of CSS and OS was 41 (IQR 6-105) months, and that of DFS was 41 (IQR 4-105) months. In the high- and low-CONUT groups, the 5-year CSS rates were 47.79% and 85.54% (P<0.001) (**Figure 2A**), the 5-year OS rates were 44.47% and 85.54% (P<0.001) (**Figure 2B**), and the 5-year DFS rates were 44.86% and 86.16% (P<0.001) (**Figure 2C**), respectively.

As shown in **Table 4**, patients with lower CONUT scores had better CSS (HR 0.197, 95% CI 0.077-0.502, P=0.001), OS (HR 0.177, 95% CI 0.070-0.446, P<0.001) and DFS (HR 0.176, 95% CI 0.070-0.444, P<0.001). In addition, Fuhrman grade was also significantly correlated with CSS, OS and DFS (P<0.01). Multivariate Cox regression analysis indicated that a low CONUT score was an independent predictor of CSS, OS and DFS (CSS: HR=0.225, 95% CI 0.067-0.749, P=0.015; OS: HR=0.201, 95% CI 0.061-0.661, P=0.008; DFS: HR=0.227, 95% CI 0.078-0.664, P=0.007). A low Fuhrman grade was an independent predictor of CSS (HR 0.192, 95% CI 0.045-0.810, P=0.025), OS (HR 0.203, 95% CI 0.049-0.842, P=0.028), and DFS (HR 0.180, 95% CI 0.048-0.669, P=0.010) (**Table 4**), while other factors, such as age, sex, hypertension, diabetes, tumor grade, histology, surgery, and CD4+ T lymphocyte count, were not associated with survival outcome.

**Table 4:** Univariate and multivariate analyses of clinicopathological parameters to predict CSS, OS and DFS in patients with HIV-related RCC.

Factors	CSS				OS				DFS			
	Univariate		Multivariate		Univariate		Multivariate		Univariate		Multivariate	
	HR (95% CI)	*P*-value	HR (95% CI)	*P*-value	HR (95% CI)	*P*-value	HR (95% CI)	*P*-value	HR (95% CI)	*P*-value	HR (95% CI)	*P*-value
**Age**												
>65	1.612 (0.548-4.745)	0.386			1.473 (0.505-4.96)	0.479			1.517 (0.520-4.423)	0.446		
≤65	1.00 (ref)				1.00 (ref)				1.00 (ref)			
**Gender**												
Male	23.809 (0.057-9932.7)	0.303			23.826 (0.075-7603.4)	0.281			2.959 (0.399-21.933)	0.289		
Female	1.00 (ref)				1.00 (ref)				1.00 (ref)			
**Fuhrman grade**												
I-II	0.086 (0.028-0.257)	**<0.01**	0.192 (0.045-0.810)	**0.025**	0.079 (0.026-0.234)	**<0.01**	0.203 (0.049-0.842)	**0.028**	0.104 (0.038-0.282)	**<0.01**	0.180 (0.048-0.669)	**0.010**
III-IV	1.00 (ref)		1.00 (ref)		1.00 (ref)		1.00 (ref)		1.00 (ref)		1.00 (ref)	
**Tumor grade**												
T1N0M0	0.757 (0.281-2.042)	0.583			0.673 (0.268-1.687)	0.398			0.823 (0.308-2.195)	0.697		
T3N0M0	1.00 (ref)				1.00 (ref)				1.00 (ref)			
**Histology**												
Clear cell	20.919 (0.000-2325088)	0.608			20.917 (0.000-1434880)	0.593			20.908 (0.000-1589742)	0.596		
Non-clear cell	1.00 (ref)				1.00 (ref)				1.00 (ref)			
**Hypertension**												
Yes	1.213 (0.360-4.087)	0.755			1.109 (0.332-3.709)	0.867			1.044 (0.312-3.491)	0.945		
No	1.00 (ref)				1.00 (ref)				1.00 (ref)			
**Diabetes**												
Yes	0.862 (0.202-3.680)	0.842			0.790 (0.186-3.352)	0.749			0.782 (0.184-3.317)	0.738		
No	1.00 (ref)				1.00 (ref)				1.00 (ref)			
**Surgery**												
RN	2.247 (0.763-6.615)	0.142			2.478 (0.849-7.229)	0.097			1.760 (0.660-4.694)	0.259		
PN	1.00 (ref)				1.00 (ref)				1.00 (ref)			
**CD4 count (cells/ul)**												
≥200	0.663 (0.225-1.951)	0.455			0.736 (0.252-2.147)	0.575			0.720 (0.247-2.099)	0.547		
<200	1.00 (ref)				1.00 (ref)				1.00 (ref)			
**CONUT score**												
<3	0.197 (0.077-0.502)	**0.001**	0.225 (0.067-0.749)	**0.015**	0.177 (0.070-0.446)	**<0.001**	0.201 (0.061-0.661)	**0.008**	0.176 (0.070-0.444)	**<0.001**	0.227 (0.078-0.664)	**0.007**
≥3	1.00 (ref)		1.00 (ref)		1.00 (ref)		1.00 (ref)		1.00 (ref)		1.00 (ref)	

Bold values indicate statistical significance in univariate and multivariate analysis which had been detailed in the “Results” section.

RCC, renal cell carcinoma; OS, overall survival; CSS, cancer-specific survival; DFS, disease-free survival; HR, hazard ratio; CONUT, controlling nutritional status; RN, radical nephrectomy; PN, partial nephrectomy.

### Compare CONUT Score With Other Biomarkers in Patients With HIV-Related RCC for Survival Prediction

The PNI and NLR values of 106 patients are also shown in **Table 2**. The ROC curve with the most sensitive and specific cutoff values of PNI and NLR is also shown in **Figure 1**. We compared the AUCs for predicting 5-year OS by CONUT, PNI and NLR. Among the prognostic factors, the CONUT has the highest AUC (0.746). The AUC score of PNI and NLR in relation to 5-year OS was 0.682 (95% CI 0.553–0.811) and 0.674 (95% CI 0.545–0.803), respectively.

## Discussion

In this study, patients with surgically treated HIV-related RCC with high CONUT scores had significantly shorter CSS, OS, and DFS than patients with low CONUT scores. Multivariate analysis further showed that the CONUT score was an independent factor influencing these survival outcomes. In addition, a low Fuhrman grade was significantly associated with survival outcomes. This study is the first to study the prognostic factors of patients with HIV-related RCC, which has certain reference significance for the surgical treatment selection of patients with HIV infection and the prognosis of patients with HIV-related RCC.

Among people living with HIV, non-HIV-related morbidity and mortality are becoming more common, and non-HIV hypotoxicity is becoming an important source of mortality (26). In recent years, there has been a significant increase in the incidence of malignant tumors in HIV-infected people because of the increased life expectancy associated with HAART, and urologists are increasingly likely to encounter HIV-infected patients with the same urinary problems as the general population (4). Strictly evaluating the surgical indications of patients with HIV-related RCC and early surgical treatment are crucial for patient prognosis. HIV mainly invades human CD4+ T lymphocytes, causing a reduction in their number and functional defects, thereby resulting in low immune function and an increasing incidence of various opportunistic infections. Although early literature on surgical outcomes in HIV-positive patients suggested an increased risk of perioperative complications (27), recent studies have shown that most procedures can be performed safely in HIV-positive patients with appropriate preoperative evaluation of CD4+ T lymphocyte count and viral load (28). Therefore, preoperative routine examination of CD4+ T lymphocytes in patients with HIV-related RCC is an aspect of evaluating whether patients can tolerate surgery, but CD4+ T lymphocytes do not serve as a good predictor of the survival prognosis of these patients; therefore, it is necessary to find prognostic predictors for patients with HIV-related RCC.

The CONUT score was determined by serum albumin, total lymphocyte counts, and total cholesterol concentration. Serum albumin is an indicator that can reflect patients’ nutritional status and is closely related to patients’ surgical tolerance and postoperative recovery. A lower level of serum albumin means the loss of immunity (29). HIV RNA levels and CD4+ T lymphocyte counts provide some prognostic information about HIV disease progression, but data published by Shruti H Mehta suggest that serum albumin levels provide more prognostic information than RNA levels and CD4 counts. Low levels of serum albumin not only reflect the general health status of HIV-infected patients but also reflect the effects of HIV on the host (30). Several studies of HIV seroepidemic cohorts have shown that low serum albumin is associated with all-cause mortality, even among individuals receiving HAART (31, 32). Sabin et al. demonstrated an independent role of serum albumin detected shortly after HIV serotransformation in all-cause mortality and a smaller but still significant role in AIDS progression. These associations were independent of CD4+ T lymphocyte count and HIV viral load (33).

Lymphocytes are believed to have antitumor ability by affecting the growth, migration, and apoptosis and inducing the cytotoxicity of tumor cells. The high density of lymphocytes reflects the immune response of tumors (34). F A Post et al. found that total lymphocyte count and CD4+ T lymphocyte count were equally good predictors of HIV infection disease progression, and severe lymphocytopenia (total lymphocyte counts <750/µl) predicted low survival and may reflect high susceptibility to opportunistic infections (35, 36). Moses R Kamya’s results showed a strong correlation between total lymphocyte counts and CD4+ T lymphocytes. Similar correlations between total lymphocyte counts and CD4+ T lymphocytes have been reported in North America, England and India (37).

Low cholesterol levels are associated with cancer outcomes. Cholesterol affects the structure and function of the membrane, such as membrane protein activity and membrane fluidity, thus affecting the ability of immunoactive cells to fight cancer cells (38). Dyslipidemia has also been observed in untreated HIV-infected patients, suggesting that HIV infection itself has deleterious metabolic effects (39). In the Swiss HIV cohort study, the use of HIV protease inhibitors was found to be associated with an increase in plasma total cholesterol (40). In the SMART study, discontinuation of HAART led to a reduction in total cholesterol concentration (41). After the initiation of HAART, lipid abnormalities in HIV patients become more obvious, and hypercholesterolemia is the most related disease (42–44). Serum total cholesterol concentration was a correlated and independent predictor of HIV RNA load, CD4+ T lymphocyte count and WHO clinical stage. In this era of testing and treatment, it is possible to use low serum total cholesterol concentration as a marker to predict the efficacy of HAART (45).

CONUT is mainly associated with malignant tumors and survival prognosis through the nutritional immune pathway. For HIV-infected patients, nutritional and immune functions are already low, and these patients have an increased risk of RCC, which is largely due to the loss of control of the oncogenic genome and the high prevalence of exposure to other carcinogens (46). Adam B Murphy et al. found that the frequency of metastatic disease in an HIV-related cohort was 2.5 times that observed in a non-HIV-related cohort, although this difference did not reach statistical significance (47). Wee Loon ONG’s study showed that a total of five HIV-related RCC patients in Australia’s statewide HIV centers underwent surgery without any perioperative complications (48). In metastatic clear cell RCC, targeted therapy or immunotherapy may interact with antiretroviral drugs to some extent (49). Annah B Layman et al. found a similar incidence, clinical presentation and outcome of RCC in HIV-infected and non-HIV-infected populations and no association between CD4+ T lymphocyte count and RCC risk at the onset of AIDS (50).

Our study has some limitations. First, the follow-up time of some patients was short, at only half a year, so it would be more meaningful to extend the follow-up time to ensure the accuracy of the results. Furthermore, the duration of treatment with antiviral drugs may also be a prognostic factor, but since most of the included patients were uncertain about the duration of HAART, no reliable data were obtained.

## Conclusion

HIV-infected patients eligible for HAART have a potentially normal life expectancy. Therefore, diseases such as RCC and other malignancies should be treated in the same way as those in non-HIV-infected patients. As it becomes increasingly possible to operate on HIV-infected patients undergoing HAART, CONUT’s role in predicting survival for HIV-related RCC is becoming increasingly important. The preoperative CONUT score not only objectively reflects the nutritional and immune statuses of the host but also is an independent predictor of CSS, OS and DFS in patients with HIV-related RCC.

## Data Availability Statement

The raw data supporting the conclusions of this article will be made available by the authors, without undue reservation.

## Ethics Statement

The studies involving human participants were reviewed and approved by Beijing You’an Hospital Affiliated to Capital Medical University. The patients/participants provided their written informed consent to participate in this study.

## Author Contributions

WX participated in manuscript preparation and writing. XH provided suggestion and edits. YZ (4th author) conceptualized, wrote, and revised manuscript. YZ (2nd author) and HW provided relevant patients data of their hospitals and offered suggestions for revising the article. All authors contributed to the article and approved the submitted version.

## Conflict of Interest

The authors declare that the research was conducted in the absence of any commercial or financial relationships that could be construed as a potential conflict of interest.

## Publisher’s Note

All claims expressed in this article are solely those of the authors and do not necessarily represent those of their affiliated organizations, or those of the publisher, the editors and the reviewers. Any product that may be evaluated in this article, or claim that may be made by its manufacturer, is not guaranteed or endorsed by the publisher.
